# Obstetrician cognitive and affective skills in a diverse academic population

**DOI:** 10.1186/s12909-016-0659-4

**Published:** 2016-05-10

**Authors:** Lynn M. Yee, William A. Grobman

**Affiliations:** Division of Maternal-Fetal Medicine, Department of Obstetrics and Gynecology, Northwestern University Feinberg School of Medicine, 250 E. Superior Street, #5-2191, Chicago, IL 60611 USA

**Keywords:** Medical education, Obstetrician cognition, Coping skills, Tolerance of ambiguity, Physician professional development

## Abstract

**Background:**

Obstetrician cognitive and affective traits have been identified to have relationships with their patients’ perinatal outcomes. The objective was to identify relationships between obstetrician demographic and practice characteristics and physician coping, self-efficacy, anxiety and ambiguity tolerance.

**Methods:**

Obstetricians at a single institution were surveyed using 5 validated scales measuring coping skills, tolerance for ambiguity, cognitive engagement and trait anxiety. Demographics and practice characteristics were assessed. Chi-square tests, t-tests, ANOVA and linear regression were used to assess relationships between physician characteristics and cognitive traits.

**Results:**

Ninety-four physicians participated. Women expressed greater proactive coping than men (*p* = 0.03) on the Proactive Coping scale. Providers with greater delivery volume expressed lower engagement in cognitive efforts (*p* = 0.03) on the Need for Cognition scale. Maternal-fetal medicine physicians demonstrated greater ambiguity tolerance (*p* < 0.01) and cognitive engagement (*p* = 0.012) than general obstetricians. Differences by specialty persisted after adjustment for potentially confounding factors.

**Conclusions:**

Practice type and specialty appeared to be related to several cognitive characteristics. It remains uncertain whether these differences are a cause or a consequence of specialty training and whether they result in differences in obstetric outcomes.

## Background

Clinical reasoning and complex medical decision making require the use of important cognitive skills. Increasingly, medical educators recognize that trainee and physician cognitive traits, including coping skills and affect, contribute to the culture of safety and the quality of clinical care [1–5]. Indeed, engagement in cognitive or critical thinking efforts, appropriate use of heuristics, analytic efficiency, and tolerance of uncertainty contribute to the quality of medical decision making [6]. Teaching, learning and practicing these clinical reasoning skills are fundamental components of medical training, particularly in fields with fast-paced clinical care and a high degree of clinical uncertainty.

However, relatively little is known about how to best promote and maintain adaptive and positive features of cognition and affect in order to optimize clinical outcomes. Greater tolerance of ambiguity, for example, has been associated with an increased chance that medical students are positively disposed toward caring for the underserved and this characteristic has been proposed as an important area for further research [7, 8]. Physician coping skills, including feelings of self-efficacy, confidence under stress, and proactive tendencies, have been associated with better obstetrical outcomes in one small study [5]. Our group’s work has investigated the associations between obstetrician cognitive and affective skills and their patients' delivery outcomes. We have demonstrated that nulliparous patients delivered by physicians with better coping skills were less likely to experience an operative vaginal delivery, whereas other “adaptive” cognitive features were associated with increased odds of chorioamnionitis and postpartum hemorrhage [9]. In addition, in a population of multiparous women with one prior cesarean delivery who were eligible for a trial of labor after cesarean, patients delivered by physicians with better coping skills were more likely to attempt a trial of labor, and patients delivered by physicians with less anxiety were more likely to achieve a vaginal birth after cesarean [10]. These data suggest that obstetrician cognitive and affective skills such as coping and anxiety may play a role in their management of labor and delivery patients [9, 10].

Notably, there is an absence of literature on the basis for differences in physician cognitive traits, including how these skills or traits may translate to clinical practice in women’s health. Since we are beginning to understand that there are relationships between physician cognitive and affective traits and their patients’ obstetrical outcomes, we propose that an important next step is to understand the provider demographic and practice characteristics that are associated with these cognitive traits. Thus, we designed this study to investigate cognitive and affective skills in a diverse population of obstetricians. The aim was to measure cognitive and affective traits, including coping skills, learner engagement, tolerance of ambiguity, and anxiety, among this population and to assess demographic and practice features associated with these traits. We hypothesized that obstetricians’ decision to sub-specialize would be associated with cognitive and affective traits.

## Methods

This is an analysis of physician-focused data collected in the above-described observational study examining the relationships between a physician’s cognitive and affective skills and their patient’s obstetric outcomes [9, 10]. The prior work focused on the independent associations between physician cognitive and affective traits and patient mode of delivery and other perinatal outcomes [9, 10]. In contrast, this analysis focuses on the obstetrician characteristics that underlie these cognitive and affective traits. In the original study, obstetricians at a single institution were recruited in person and signed written, informed consent prior to their participation in the survey study. All providers at this large, university institution practice in a teaching hospital setting and hold university appointments; some are considered full-time academic faculty whereas others are contributed services faculty. All clinicians are responsible for teaching trainees, and trainees are involved in all aspects of patient care. At the time of this survey, 115 obstetricians provided obstetrical care at this institution. Of these, 9 were maternal-fetal medicine [MFM] subspecialists, and the remainder were general obstetrician/gynecologists. No full-time laborists were in practice at this institution. Mid-level providers were not eligible for study participation.

Obstetricians enrolled completed a survey that assessed demographic data, including physician age, gender, ethnicity, practice specialty (general obstetrics versus maternal-fetal medicine), number of years since completion of training, and number of deliveries per year. Detailed personal demographic information collected was relatively limited in order to protect participant confidentiality for the primary analysis. Participants then completed five validated psychometric scales, described below and summarized in Table 1. In the prior studies, the relationships between participant scores on the scales of cognitive and affective traits and their patients’ delivery outcomes were assessed; in this study, survey information on physician participant demographic and practice characteristics were the focus of investigation, rather than patient outcomes.

**Table 1:** Cognitive and affective scales used to assess physician traits

Instrument	Constructs	Number of items
Reflective Coping (RC) sub-scale of the Proactive Coping Inventory [11, 12]	• coping in the setting of stress and distress • self-efficacy • affect and proactive attitude	11
Proactive Coping (PC) sub-scale of the Proactive Coping Inventory [11, 12]	• proactive goal attainment/orientation • self-confidence • self-regulatory cognition & behavior	14
Multiple Stimulus Types Ambiguity Tolerance - II (MSTAT-II) [5, 13, 14]	• tolerance for ambiguity • degree of comfort with uncertainty and/or complexity • receptiveness to change	13
Need for Cognition (NFC) [5, 15]	• learner motivation • tendency to engage in and enjoy cognitive efforts • positive self-esteem, successful adaptive decision-making • reflects individual affect in processing cognitive information • low NFC indicates social anxiety and difficulty with decision making	18
State-Trait Anxiety Inventory (STAI) - Trait component [16]	• stable individual tendencies toward anxiety in a range of threatening situations • measure of affect	20

The Reflective Coping (RC) sub-scale of the Proactive Coping Inventory (PCI), which assesses self-efficacy, is an 11-item questionnaire assessing the range of behaviors used to manage challenging situations [11]. It is considered a good measure of cognition, as it describes “simulation and contemplation about a range of possible behavioral alternatives by comparing their perceived effectiveness” [12]. Higher scores on the RC scale are associated with a higher perceived self-efficacy [5]. The Proactive Coping (PC) sub-scale of the PCI assesses proactive goal attainment, desire to succeed and self-confidence [11]. This scale is a 14-item scale that “combines autonomous goal setting with self-regulatory goal attainment cognitions and behavior” [11].

The Multiple Stimulus Types Ambiguity Tolerance - II (MSTAT-II) scale is an updated, shorter (13-item) version of the Multiple Stimulus Types Ambiguity Tolerance-I, which assesses tolerance for ambiguity [5, 13]. Tolerance for ambiguity reflects an individual’s range of responses to and degree of comfort with uncertainty and/or complexity. The MSTAT-II is felt to be a reliable assessment of an individual’s “cognitive orientation toward several types of ambiguous stimuli” [14].

The 18-item Need for Cognition (NFC) scale reflects an individual’s tendency to “engage in and enjoy cognitive efforts,” and is felt to reflect individual affect in processing cognitive information [15]. A high NFC score is felt to reflect positive self-esteem and more successful adaptive decision-making, including greater accuracy and consistency of decision-making [5]. A low NFC score suggests greater social anxiety and difficulty with decision-making [5].

The State-Trait Anxiety Inventory (STAI) is a two-part scale measuring both trait and state anxiety. Each component includes 20 items for trait anxiety (i.e., stable individual tendencies toward anxiety) and 20 items for state anxiety (i.e., transitory anxiety at the time of measurement) [16]. Higher scores indicate greater anxiety. Results of the STAI scale are considered a measure of affect. In this study, we only measured trait anxiety, as the state anxiety results were felt to be less relevant to the study’s objectives.

Each scale was scored in the usual technique per original scale instructions. These scales are not indended to be analyzed dichotomously (ie, there are no designated cut-points for “normal” or “abnormal” traits). Thus, all scores were analyzed as continuous variables, since the goal of this analysis was to assess differences between providers rather than whether or not the traits themselves are adaptive or maladaptive. Provider characteristics were assessed using descriptive statistics. Demographic characteristics between general obstetrician/gynecologists and maternal-fetal medicine specialists were compared using chi-squared tests. Scores for each scale were then analyzed for differences between demographic characteristics, specialty type, number of years in practice, and delivery volume, using t-tests and one-way analysis of variance (ANOVA), as appropriate. Assessment of normal distribution of variables was performed, and confirmed that scores for the RC, PC, MSTAT, and NFC were normally distributed, and thus parametric tests were utilized. The results for the STAI were not normally distributed, and thus non-parametric tests (Wilcoxon rank-sum or Kruskal-Wallis tests) were performed. Multivariable linear regression analyses were then carried out to determine the independent effect of physician practice type (general obstetrician/gynecologist versus maternal-fetal medicine) on pyschometric scores; demographic factors with a *p* < 0.1 on bivariable analysis (gender and delivery volume) were included as potential confounders in the regression model. All tests were two-tailed with *P* < .05 used to define statistical significance. Analyses were performed with Stata 11 (Stata, Inc., College Station, TX). The Northwestern University Institutional Review Board approved this study and all participants provided written informed consent.

## Results

Of the 115 eligible providers, 94 (82 %) participated in this study. Eight-five participants were general obstetrician/gynecologists and 9 were maternal-fetal medicine subspecialists; all members of the MFM group at this institution participated. There were no differences in demographic characteristics between generalists and MFMs. Approximately 73 % of participants were between ages 31–50 and 74 % were female. Approximately 70 % were self-reported to be Caucasian, 7 % African American, 3 % Hispanic, and 19 % Asian American. Approximately 21 % had been in practice for 5 or fewer years, 53 % between 6 and 20 years, and 26 % greater than 20 years. The median number of deliveries per year was 120 (interquartile range 100–150).

There were few differences in cognitive scale scores based on demographic characteristics. Mean scores for each scale by demographic characteristics are shown in Table 2. Scores for the RC, PC, MSTAT, and NFC scales were normally distributed. The mean RC score was 35.8 (SD 4.0, range 25–44). There were no differences in RC score by age, race/ethnicity, gender, or delivery volume. Mean PC score was 44.6 (SD 4.8, range 30–54). There were no differences in PC score by age, race/ethnicity, or delivery volume. However, women were noted to have slightly higher PC scores than men (45.2 vs 42.8, *p* = 0.034, *t*-test). Mean MSTAT score was 61.6 (SD 9.9, range 35–87). There were no differences in MSTAT score by age, race/ethnicity, gender, or delivery volume. Mean NFC score was 66.6 (SD 10.3, range 43–90). There were no differences in NFC score by age, race/ethnicity, or gender. However, NFC score was noted to be lower for providers with the greatest number of deliveries per year (*p* = 0.032, ANOVA). Mean STAI was 32.9 (SD 8.0, range 21–60). There were no differences in STAI score by age, race/ethnicity, gender or delivery volume. There were no differences in any scores by number of years in practice.

**Table 2:** Obstetrician cognitive trait scores by demographic and practice characteristics

	RC^a^	PC^b^	MSTAT^c^	NFC^d^	STAI^e^
	Mean (SD)	Mean (SD)	Mean (SD)	Mean (SD)	Mean (SD)
Age					
25-30 31–40 41–50 51–60 >61	36.5 (0.7) 35.6 (4.3) 35.5 (4.3) 36.4 (3.4) 37.3 (2.7)	45.0 (4.2) 46.1 (4.5) 43.1 (5.0) 45.2 (4.9) 43.3 (3.8)	51.0 (7.1) 60.6 (11.4) 61.3 (8.2) 63.4 (6.6) 66.7 (17.1)	67.0 (7.1) 66.1 (10.4) 65.9 (9.7) 67.9 (10.6) 69.7 (15.0)	34.5 (3.5) 32.7 (7.2) 33.9 (9.4) 34.1 (7.0) 25.7 (4.8)
Ethnicity					
Caucasian African American Hispanic Asian American	35.7 (4.2) 35.1 (4.0) 37.0 (6.1) 36.4 (3.3)	44.4 (4.9) 46.9 (4.5) 50.0 (5.2) 43.6 (4.2)	62.3 (9.5) 62.3 (16.9) 64.0 (10.4) 58.1 (8.1)	66.7 (11.0) 62.3 (10.6) 75.7 (12.7) 66.3 (6.4)	32.8 (8.0) 30.7 (4.4) 27.7 (4.9) 35.4 (9.1)
Gender					
Male Female	36.1 (3.1) 35.7 (4.3)	42.8 (5.7)* 45.2 (4.4)	62.7 (10.5) 61.2 (9.8)	66.1 (10.2) 66.8 (10.4)	33.6 (9.3) 32.7 (7.6)
Years in practice					
≤5 6–10 11–20 >20	35.2 (4.5) 36.6 (4.0) 35.4 (4.2) 36.5 (3.3)	45.0 (4.3) 46.5 (4.2) 43.5 (4.8) 44.4 (5.4)	58.0 (10.7) 60.6 (9.9) 62.4 (9.4) 64.1 (9.9)	64.2 (11.8) 67.8 (7.8) 66.0 (10.1) 68.7 (11.0)	31.9 (6.6) 35.8 (8.4) 32.3 (9.0) 32.8 (7.5)
Number of deliveries per year					
≤50 51–100 101–150 ≥150	40.0 (2.2) 36.4 (4.1) 35.4 (4.0) 35.5 (3.7)	43.3 (6.7) 44.0 (5.5) 45.2 (4.3) 44.6 (4.6)	63.0 (16.2) 64.5 (12.0) 60.7 (7.8) 58.4 (8.3)	73.8 (11.7)** 70.2 (11.0) 64.4 (9.4) 64.1 (9.1)	36.0 (7.7) 34.9 (7.8) 31.2 (7.3) 32.8 (9.3)

Data presented as mean (SD) for raw scores. For all scores except STAI, higher values indicate more’"adaptive" traits

*P = 0.034 (*t*-test)

**P = 0.032 (ANOVA)

^a^Reflective Coping scale measures self-efficacy and coping in the setting of stress

^b^Proactive Coping scale measures proactive goal attainment, self-confidence, and self-regulatory behavior

^c^Multiple Stimulus Types Ambiguity Tolerance scale measures tolerance of ambiguity, degree of comfort with uncertainty, and receptiveness to change

^d^Need for Cognition scale measures learner motivation, engagement with cognitive efforts, and adaptive decision making

^e^Trait component of the State-Trait Anxiety Inventory measures stable tendencies toward anxiety and is a measure of affect. Lower score reflects less trait anxiety

Differences in scores based on specialty type were examined. In comparing specialty type, MFM physicians scored higher in the MSTAT and NFC scales, indicating both a higher tolerance of ambiguity and greater learner engagement (Table 3). There were no differences in scores by specialty for the coping or anxiety scales. Using multivariable regression to assess the independent association of subspecialty and scores on each scale, adjusting for physician gender and number of deliveries per year, we found the MSTAT and NFC scores remained significantly higher for MFM physicians.

**Table 3:** Differences in cognitive traits between general obstetricians and maternal-fetal medicine specialists

	Bivariable analysis			Multivariable linear regression^a^	
	MFM mean (SD)	General Ob/Gyn mean (SD)	*p*-value	Effect estimate (95 % CI)	*p*-value
Reflective coping^b^	34.8 (3.4)	36.0 (4.1)	0.41	−1.4 (−4.2 – 1.4)	0.31
Proactive coping^c^	43.8 (5.8)	44.7 (4.7)	0.60	−0.6 (−4.0 – 2.7)	0.72
Tolerance of ambiguity^d^	69.7 (10.2)	60.7 (9.6)	<0.01	8.3 (1.6 – 15.0)	0.016
Need for cognition^e^	74.8 (10.5)	65.8 (10.0)	0.012	8.3 (1.5 – 15.1)	0.018
Trait anxiety^f^	31.3 (7.8)	33.1 (8.1)	0.45	−2.3 (−7.9 – 3.4)	0.43

*MFM* maternal-fetal medicine

^a^Score reflects the difference in score for MFMs compared to generalists, adjusting for gender and number of deliveries per year. For all traits except trait anxiety, higher scores indicate more “adaptive” traits

^b^Reflective Coping scale measures self-efficacy and coping in the setting of stress

^c^Proactive Coping scale measures proactive goal attainment, self-confidence, and self-regulatory behavior

^d^Multiple Stimulus Types Ambiguity Tolerance scale measures tolerance of ambiguity, degree of comfort with uncertainty, and receptiveness to change

^e^Need for Cognition scale measures learner motivation, engagement with cognitive efforts, and adaptive decision making

^f^Trait component of the State-Trait Anxiety Inventory measures stable tendencies toward anxiety and is a measure of affect

## Discussion

Understanding physician cognitive traits is critical to professional development and education, as these skills may affect both clinical decisions and learner success. This study examined features of obstetrician decision making, coping skills, engagement in cognitive effort, ambituity tolerance, and anxiety in a large, diverse population of practicing obstetrician/gynecologists. We investigated the relationships between physicians’ personal and specialty characteristics and their scores on five established, validated scales of cognitive skills and affect. While few meaningful differences were identified based on demographic characteristics, we identified several differences in cognitive traits based on physician specialty. These findings may have clinical significance, as our prior work has demonstrated that there is an association between physician cognitive and affective traits and their patients’ obstetrical outcomes [9, 10]. Yet, no prior work has examined features underlying or contributing to these cognitive traits. Given the element of uncertainty occurring in the care of obstetrical patients, these findings may have implications for patient care as well as important applications to medical education and professional development.

In this observational study, we identified physicians who chose careers in the subspecialty of maternal-fetal medicine scored higher on two scales: the Multiple Stimulus Types Ambiguity Tolerance-II scale and the Need for Cognition scale. These results suggest that academic obstetricians with careers in maternal-fetal medicine have greater comfort with uncertainty and complexity and may have better coping in the setting of ambiguous stimuli. Similarly, these physicians also appear to have more adaptive decision making, greater self-esteem, and increased learner engagement, as reflected in the NFC scale. Learner engagement may be reflected in behaviors such as consulting the primary literature when faced with indecision, seeking out guidelines and medical literature to further clinical knowledge, promoting the learning and teaching process with peers and trainees, and performing adaptive clinical decision making that incorporates evolving situations and clinical knowledge. It is important to note that these traits exist on a continuum and the raw scores do not suggest that either type of physician has an “adaptive” versus “maladaptive” trait, but merely that there is a difference between the generalist and subspecialist obstetricians. Further research investigating trainees as well as more experienced clinicians is required to better understand whether these differences are a result of subspecialty training or are an underlying reason for career choice.

In addition, providers with the greatest delivery volume were noted to have lower scores on the NFC scale, which assesses engagement with learning and critical thinking. It is not clear if high volume obstetricians chose such practice styles due to this cognitive style or if a busy practice resulted in less time for engagement in reflective decision making and in-depth learning and processing. Interestingly, there were no other differences in these traits by other demographic features aside from the slightly higher proactive coping scores noted for women compared to men. The lack of difference in scores based on number of years in practice suggests cognitive skills may be somewhat intrinsic and less dependent on experience or career stage.

The primary applications for this study are in the realm of medical education and professional development. While we do not know whether individual cognitive traits measured herein are the reason for a career choice or a consequence of training in a specific manner, it is possible training and education focusing on coping skills and decision making could enhance physician professional development and quality of decisions. Physician learning does not stop with graduation from medical school or residency; instead, physicians are expected to continuously engage in thinking and learning, reflect on clinical decisions, and refine their practice patterns in response to their goals and outcomes. Yet, these data would suggest some obstetricians engage in these skills differently than others, and these differences may translate to differences in clinical care. It is increasingly clear that cognitive biases and cognitive efforts have relationships to patient care and clinical outcomes [1, 6, 17, 18]. One important application of this research is in learning how to provide physicians ongoing education and feedback about their decision making and copking skill set with the ultimate goal of improving patient care. Active engagement in “cognitive debiasing” education is one of many potential ways to help improve clinical reflection and problem solving skills [17, 19]. Finally, an additional application is in medical career counseling; it is possible that an improved understanding of one’s cognitive strengths may aid in choosing specialties and subspecialties that are best suited for each individual’s skills.

There are several limitations to consider. First, this study is limited to the obstetricians in practice at a single, large, university-based institution. These data may not be readily generalizable to other practice settings, such as those without residents or a university presence. However, the majority of providers at our institution completed the study and these providers represent a diverse population of ages, practice types, training backgrounds, ethnic groups, and years in practice. Second, in order to protect the identities of participants, physician demographic data was collected in as little detail as possible; for example, rather than asking exact age and number of years in practice, which could make a participant identifiable after also asking gender and race/ethnicity, such information was asked in ranges. This intentional strategy may have limited the granularity of the findings. Third, the subspecialist sample in this study was small. Although all 9 maternal-fetal medicine subspecialists in this institution completed the survey, this small sample size warrants further investigation in larger studies beyond a single university. Further, non-responders may have introduced selection bias, or providers may have felt social pressure to choose what they felt to be socially desirable responses on their survey. However, if these biases exist, we would expect them to bias toward the null hypothesis. Finally, an additional limitation of this exploratory study is the performance of multiple comparisons, which may increase risk of Type I error; however, in the setting of a hypothesis-generating study, the risk of Type I error is felt to be acceptable in exchange for reducing Type II error, as this small study is intended to raise questions for future work.

## Conclusions

Obstetrician tolerance of ambiguity and need for cognition appear to be related to features of career choice, with subspecialist obstetricians demonstrating different cognitive traits from general obstetricians. While we do not know whether intrinsic cognitive traits were the foundation for specialty choices or whether these cognitive traits developed during training, these questions deserve further investigation. Clinical reasoning is a cognitive effort requiring constant reevaluation and familiarity with multiple cognitive and affective biases. Obstetrical care in particular is a field with multiple areas of uncertainty in which coping skills, ambiguity tolerance, and decision making skills have relevance to patient outcomes. Further investigative work regarding the relationships between learner motivation, intrinsic cognitive traits, and patient care in the setting of obstetrics and gynecology appears warranted.

## Approval

This study was approved by the Northwestern University Institutional Review Board (STU00070810).

## Consent for publication

NA.

## Availability of data

The participant survey data supporting the conclusions of this manuscript are not publicly available for participant privacy protection.

## Abbreviations

MFM: Maternal-fetal medicine
MSTAT: Multiple Stimulus Types Tolerance of Ambiguity
NFC: Need for Cognition
PC: Proactive Coping
PCI: Proactive Coping Inventory
RC: Reflective Coping
STAI: State-Trait Anxiety Inventory

## References

[1] Croskerry P, Abbass A, Wu AW (2008). How doctors feel: Affective issues in patients’ safety. Lancet.

[2] Croskerry P, Abbass A, Wu AW (2010). Emotional influences in patient safety. J Patient Saf.

[3] McConnell MM, Eva KW (2012). The role of emotion in the learning and transfer of clinical skills and knowledge. Acad Med.

[4] Wetzel CM, Black SA, Hanna GB, Athanasiou T, Kneebone RL, Nestel D, Wolfe JH, Woloshynowych M (2010). The effects of stress and coping on surgical performance during simulations. Ann Surg.

[5] Dunphy BC, Cantwell R, Bourke S, Fleming M, Smith B, Joseph KS, Dunphy S (2010). Cognitive elements in clinical decision-making: Toward a cognitive model for medical education and understanding. Adv Health Sci Educ Theory Pract.

[6] Croskerry P (2013). From mindless to mindful practice: Cognitive bias and clinical decision making. N Engl J Med.

[7] Wayne S, Dellmore D, Serna L, Jerabek R, Timm C, Kalishman S (2011). The association between intolerance of ambiguity and decline in medical students’ attitudes toward the underserved. Acad Med.

[8] Luther VP, Crandall SJ (2011). Commentary: Ambiguity and uncertainty: neglected elements of medical education curricula?. Acad Med.

[9] Yee LM, Liu LY, Grobman WA. The relationship between obstetricians’ cognitive and affective traits and their patients’ delivery outcomes. Am J Obstet Gynecol. 2014;211(6):692. e1–6. 10.1016/j.ajog.2014.06.003 24907699

[10] Yee LM, Liu LY, Grobman WA (2015). Relationship between obstetricians’ cognitive and affective traits and delivery outcomes among women with a prior cesarean. Am J Obstet Gynecol.

[11] Greenglass ER, Schwarzer R, Jakubiec S, Fiksenbaum L, Taubert S (1999). The Proactive Coping Inventory (PCI): A Multidimensional research instrument. Paper presented at the 20th International Conference of the STAR (Stress and Anxiety Research Society): 9/3/2012 1999; Cracow, Poland.

[12] Greenglass ER, Frydenberg E (2002). Proactive Coping. Beyond coping: Meeting goals, vision, and challenges.

[13] McLain DL (1993). The MSTAT-I: a new measure of an individual’s tolerance for ambiguity. Educ Psychol Meas.

[14] McLain DL (2009). Evidence of the properties of an ambiguity tolerance measure: the Multiple Stimulus Types Ambiguity Tolerance Scale-II (MSTAT-II). Psychol Rep.

[15] Cacioppo JT, Petty RE, Kao CF (1984). The efficient assessment of need for cognition. J Pers Assess.

[16] Spielberger CD, Gorsuch RL, Lushene R, Vagg PR, Jacobs GA (1983). Manual for the State-Trait Anxiety Inventory.

[17] Croskerry P (2003). The importance of cognitive errors in diagnosis and strategies to minimize them. Acad Med.

[18] Pauker SG, Wong JB (2010). How (should) physicians think? A journey from behavioral economics to the bedside. JAMA.

[19] Trowbridge RL, Dhaliwal G, Cosby KS (2013). Educational agenda for diagnostic error reduction. BMJ Qual Saf.

